# Experimental strategy for the preparation of adsorbent materials from torrefied palm kernel shell oriented to CO_2_ capture

**DOI:** 10.1007/s11356-024-32028-3

**Published:** 2024-02-13

**Authors:** Marlon Cordoba-Ramirez, Farid Chejne, Jader Alean, Carlos A. Gómez, África Navarro-Gil, Javier Ábrego, Gloria Gea

**Affiliations:** 1 Mechanical Engineering Program – DESTACAR Research Group, Faculty of Engineering, Universidad de La Guajira, km 3+354 via Maicao, 440001 Riohacha, Colombia; 2 Department of Processes and Energy – Applied Thermodynamics and Alternative Energies Research Group, Faculty of Mines, Universidad Nacional de Colombia Sede Medellín, Cra. 80 No 65 – 223, 050034 Medellín, Colombia; 3 Thermochemical Processes Group (GPT), Aragon Institute for Engineering Research (I3A), Universidad de Zaragoza, Edificio I+D, C/Mariano Esquillor s/n, 50018 Zaragoza, Spain

**Keywords:** Torrefaction, Pyrolysis, Activated carbon, Biochar, Porous structure, CO_2_ adsorption

## Abstract

In this study, an experimental strategy to obtain biochar and activated carbon from torrefied palm kernel shell as an efficient material for CO_2_ removal was evaluated. Biochar was obtained by slow pyrolysis of palm kernel shell at different temperatures (350 °C, 550 °C, and 700 °C) and previously torrefied palm kernel shell at different temperatures (220 °C, 250 °C, and 280 °C). Subsequently, activated carbons were prepared by physical activation with CO_2_ from previously obtained biochar samples. The CO_2_ adsorption capacity was measured using TGA. The experimental results showed that there is a correlation between the change in the O/C and H/C ratios and the functional groups –OH and C=O observed via FTIR in the obtained char, indicating that both dehydration and deoxygenation reactions occur during torrefaction; this favors the deoxygenation reactions and makes them faster through CO_2_ liberation during the pyrolysis process. The microporous surface area shows a significant increase with higher pyrolysis temperatures, as a product of the continuous carbonization reactions, allowing more active sites for CO_2_ removal. Pyrolysis temperature is a key factor in CO_2_ adsorption capacity, leading to a CO_2_ adsorption capacity of up to 75 mg/g_CO2_ for biochar obtained at 700 °C from non-torrefied palm kernel shell (Char700). Activated carbon obtained from torrefied palm kernel shell at 280 °C (T280-CHAR700-AC) exhibited the highest CO_2_ adsorption capacity (101.9 mg/g_CO2_). Oxygen-containing functional groups have a direct impact on CO_2_ adsorption performance due to electron interactions between CO_2_ and these functional groups. These findings could provide a new experimental approach for obtaining optimal adsorbent materials exclusively derived from thermochemical conversion processes.

**Supplementary Information:**

The online version contains supplementary material available at 10.1007/s11356-024-32028-3.

## Introduction

Growing concern about replacing fossil fuels and conventional polluting energy sources has generated interest in exploring new alternatives from renewable sources. Consequently, there has been a surge in the research and development of technologies aimed at mitigating pollutants such as CO_2_, CH_4_, N_2_O, and others, which contribute to atmospheric pollution and climate change. Rising global energy demand has been a significant contributor to the increased concentration of CO_2_ in the atmosphere. Consequently, various capture and storage technologies have been proposed as viable alternatives to reduce these emissions and prevent their release into the atmosphere.

Porous adsorbent materials have emerged as a highly promising solution for removing pollutants in gas and aqueous phases (El-Metwaly et al. 2022; Al-Hazmi et al. 2022; Almahri et al. 2023a). Among these materials, biochar and activated carbon have garnered significant attention for several reasons, including their abundance of renewable raw materials, making them particularly appealing (Li et al. 2008; Zhang et al. 2017; Cheng and Li 2018; Ranguin et al. 2021), and their surface and textural properties, such as high surface areas, diverse pore sizes, and surface chemistry (Sethupathi et al. 2017; Gasquet et al. 2020; Scapin et al. 2021; Zhu et al. 2021). These materials can be obtained from agro-industrial lignocellulosic biomass residues, which can be transformed by thermochemical processes such as torrefaction and slow pyrolysis. Several agricultural wastes such as coffee husk (Mukherjee et al. 2022), coffee grounds (Laksaci et al. 2017; Chen et al. 2021), rice husk and rice straw (Chen et al. 2017a; Zhang et al. 2020; Gao et al. 2021), coconut shell (Arena et al. 2016; Gongxiang et al. 2022; Ighalo et al. 2023), and palm kernel shell (Uemura et al. 2015; Md Zaini and Hassan 2018; Adilla Rashidi et al. 2021) have been proposed as precursors for carbon materials obtained by pyrolysis and torrefaction. Among these, palm kernel shell has gained special attention due to its availability (1.6 million ton/year of palm residues in Colombia) (Marrugo et al. 2016), and its physicochemical properties such as high lignin content, high carbon content, and high volatile matter content (Marrugo 2015), which make it suitable for thermochemical conversion through torrefaction and pyrolysis to obtain solid carbon materials (Vega et al. 2019).

Different studies have focused their attention on the use of carbon materials from lignocellulosic wastes in the removal of these gases at different concentrations (Sharma et al. 2021; Lai et al. 2021; Fernandes et al. 2021; Ro et al. 2021; Aghel et al. 2022), considering important parameters such as the porosity developed, the functional groups present on the surface, and even additives that improve the performance during the process. Ma et al. (2021) conducted a comparative study of the performance of biochar samples for both CO_2_ and H_2_S removal in biogas streams. This study presented relevant results regarding the effect of N-containing functional groups on the surface of biochar samples, indicating their beneficial effect on solid yield, porous structure, and CO_2_ retention.

To improve the performance of biomass and the final product of pyrolysis, some pretreatments are usually required before its conversion. Currently, pretreatment of biomass includes impregnation with acids and bases (Ahmed et al. 2016), impregnation with metals (Cai et al. 2016; Cho et al. 2017; He et al. 2018), and thermochemical processes such as torrefaction (Xu et al. 2018; Zeng et al. 2019; Sibiya et al. 2021). This process consists of partial pyrolysis of the biomass carried out in a short range of temperatures between 200 and 300 °C in an inert environment to remove moisture and some volatiles contained in the biomass (Nhuchhen et al. 2014). Some advantages of torrefaction include improved thermal stability, equilibrium moisture absorption, heating value, and biomass reactivity (Granados et al. 2022). In turn, it is possible to obtain bio-oils with a higher content of phenolic compounds derived from lignin (Park et al. 2013; Pelaez-Samaniego et al. 2014).

Despite the existing knowledge about the benefits of torrefaction to obtain liquids and gasses, little attention has been paid to the changes in biochar, including its porous structure, chemical composition, and performance in adsorption applications and to obtain activated carbon. Zhang et al. (2016) investigated the effect of torrefaction on the yield and quality of pyrolyzed biochar with applications in the production of activated carbon. These authors propose the route “torrefaction–pyrolysis–activation (chemical)” to evaluate the yields of char, liquids, and gasses as well as the textural properties of activated carbon. They found that torrefaction improved the properties of the precursor (rice husk) as a fuel and had significant effects on the fixed carbon content of char obtained from pyrolysis, which is reflected in the final characteristics of activated carbon. However, it is unclear what influence the activating agent (NaOH) could have on the increase in surface area, which is initially due to the increase in torrefaction temperature. Chen et al. (2017b) studied the influence of torrefaction on the physicochemical characteristics of char during the pyrolysis of rice straw washed with pure water and unwashed. The results show that higher torrefaction temperatures result in higher solid yields after pyrolysis. This is mainly due to the increase in the fraction of fixed carbon that occurs because the torrefaction temperature increases, thus causing severe carbonization of some macromolecules with increasing temperature during the process. Similarly, Zheng et al. (2013) proposed a simplified mechanism showing the effect of torrefaction on rapid cellulose pyrolysis. During torrefaction, the cellulose is depolymerized to form active cellulose, which successively undergoes polymerization and crosslinking reactions that lead to the formation of char. After a more severe pyrolysis, the crosslinking and the obtained char result in more solid structures, causing greater production of active carbon sites, which makes biochar prone to react with the molecules of the gasifying agent during activation (Wang et al. 2018). They also found, as did Zhang et al. (2016), that the surface area decreases with increasing torrefaction temperature after the pyrolysis process; this phenomenon can be attributed to changes in the volatile material content between the virgin and torrefied biomasses. The results of the proximate analysis indicate a lower volatile material fraction at higher torrefaction temperatures. Although the reported literature has studied in detail the impact of torrefaction on biochar and activated carbon properties and composition that make it a promising pretreatment, there is still a lack of information related to the performance of these materials obtained from torrefied biomass in CO_2_ capture applications, which could be beneficiated with the structural changes promoted by the torrefaction process. In addition, the reported literature on this is limited to a few agricultural wastes (rice husk and rice straw). Therefore, a detailed study of biochar and activated carbon obtained from torrefied palm kernel shells for CO_2_ capture is necessary.

This study aims to assess the impact of torrefaction as a pretreatment on palm kernel shells to produce biochar and activated carbon, which serve as efficient materials for CO_2_ capture. This research encompasses a compilation of experimental findings that examine alterations in thermal stability through thermogravimetric analysis, elemental composition, and porous structure via N_2_ and CO_2_ adsorption. In addition, functional groups were analyzed using FTIR spectroscopy for both raw and torrefied palm kernel shells and their respective biochar and activated carbon. The acquired results offer a comprehensive understanding of the thermochemical conversion effects on palm kernel shells, encompassing torrefaction and slow pyrolysis at various temperatures and the combination of these processes. These findings reveal correlations between the elemental and chemical compositions of functional groups, highlighting significant influences on deoxygenation reactions during torrefaction. Such reactions may alter potential active sites on char and activated carbon samples, consequently affecting the performance of CO_2_ capture. Furthermore, this study provides a crucial comparative analysis of porous structural changes across a spectrum of torrefaction and pyrolysis temperatures, extending to both char and activated carbon samples. The results derived from this experimental approach also open new avenues for optimizing the production process of carbonaceous adsorbent materials (biochar and activated carbon) designed for CO_2_ capture.

## Materials and methods

### Char and activated carbon preparation

Palm kernel shell obtained from Norte de Santander (Colombia) was used. The material was dried in a furnace at 110 °C for 24 h to remove moisture. The dry biomass was grinded using a homemade hammer mill, reducing the particle size up to 1– 2 mm. Palm kernel shells were labeled RawPKS.

Torrefied palm kernel shell and biochar were prepared at different temperatures in a fixed-bed horizontal oven. For each experiment, 10 g of palm kernel was loaded into a metal spoon and subsequently placed in the center of the quartz tube used as reactor. Prior to the test, the inside of the quartz tube was purged with a constant flow of 100 mL/min STP of nitrogen for 10 min to remove all the remaining air and oxygen. Finally, the oven was heated to the desired temperature as indicated below. For torrefied palm kernel shell preparation, temperatures of 220 °C, 250 °C, and 280 °C were selected. These samples were labeled as T220, T250, and T280, indicating that they were obtained through torrefaction at the proposed temperatures. Biochar prepared from raw palm kernel shell was obtained at temperatures of 350 °C, 550 °C, and 700 °C; these samples were labeled Char350, Char550, and Char700, indicating that these samples were obtained at the proposed temperatures. The specified temperatures (for torrefaction and pyrolysis process) were chosen with precision, considering previous reports affirming significant structural changes within the biomass at these temperature ranges, primarily driven by transformative reactions involving its major constituents—hemicellulose, cellulose, and lignin (Jia and Lua 2008a; Yang et al. 2016; Granados Morales 2017). To analyze the effect of previous torrefaction, torrefied palm kernel shells were also pyrolyzed at a temperature of 700 °C, considering the properties and porous structure of biochar obtained at this temperatures from original palm kernel shell. The samples obtained from pyrolysis of torrefied palm kernel shells were labeled as T220-Char700, T250-Char700, and T280-Char700, indicating that they are biochar obtained at 700 °C from torrefied palm kernel shell at 220 °C, 250 °C, and 700 °C.

For the torrefaction and pyrolysis experiments, a heating rate of 10 °C/min was used. A nitrogen flow of 100 mL/min STP was used in all experiments with a residence time of 1 h. At the end of each process, samples were cooled by removing the metal spoon from the oven but keeping it within an excess section of the quartz tube with a continuous flow of nitrogen for 2 h until completely cooled, to avoid spontaneous combustion.

Subsequently, activated carbon was obtained from the biochar samples. The activated carbon was obtained from physical activation in CO_2_ atmosphere at 850 °C. For this, the same experimental device was used, programing the heating of the oven at 850 °C with a heating rate of 10°C/min in N_2_ atmosphere with the sample placed in the excess section of the quartz tube. After reaching the required temperature, the gas was changed from N_2_ to CO_2_, and biochar was introduced into the heating zone to start the activation process. After reaching the required temperature, the gas was changed from N_2_ to CO_2_ and biochar was introduced for 6 h to perform the activation process; the CO_2_ flow was set at 100 mL/min. At the end of the process, the sample is cooled in a nitrogen atmosphere to prevent spontaneous combustion. Activated carbon obtained from each biochar sample can be identified with the sample label followed by AC (for example, Char700-AC indicates that it is the activated carbon prepared from biochar obtained from pyrolysis at 700 °C).

### Characterization

The elemental analysis of the obtained biochar was performed according to the standard on an Exeter CE-440 Elemental Analyzer. The oxygen content was calculated by difference with the percentage of C, H, N, and ash content.

The thermal performance was evaluated by thermogravimetric analysis (TGA) on a Linseis STA PT 1600 thermogravimetric scale. The experiments were performed in a N_2_ atmosphere with a continuous flow of 50 mL/min and a heating rate of 10 °C/min. The samples were heated between room temperature (approx. 20 °C) and 700 °C.

The textural parameters of the samples were characterized by N_2_ isotherms at 77 K and CO_2_ isotherms at 273 K in a surface area and porosity analyzer system (Micromeritics TrisTar II Plus). Prior to the adsorption measurements, the samples were degassed at 250 °C for 24 h to release the moisture contained in the pores. Nitrogen adsorption isotherms were measured in a relative pressure range (*p*/*p*_0_) between 0.04 and 0.99. The BET surface area was calculated from the nitrogen adsorption isotherms using the Brunauer–Emmett–Teller equation (BET), assuming a sectional area of 0.162 nm for N_2_ molecule (Brunauer et al. 1938). The total pore volume was calculated from the amount of nitrogen adsorbed at *p*/*p*0 = 0.99 converted to its liquid volume. The mean pore diameter was calculated from the ratio 4000Vt/*A*_(BET)_. The surface area and micropore volume were calculated using Dubinin Raduskevich’s model. The pore size distribution was calculated using non-local density functional theory (NLDFT).

Morphological analysis of the samples was performed using FE-SEM microscopy technique. To this end, a Carl Zeiss® Merlin FE-SEM microscope was used by applying an accelerated voltage of 5 kV. Before analysis, the samples were impregnated with a gold coating to ensure good conductivity of the atoms on the surface.

The chemical composition of the surface of the biochar samples was characterized by attenuated total reflection Fourier transform infrared spectroscopy (ATR-FTIR) using an Agilent Cary 600 spectrometer with a resolution of 4 cm^−1^ in the wavenumber range between 4000 and 400 cm^−1^ (mid-IR region). These tests were performed in triplicate for each char sample. The absorbance results are presented as the average of each test corresponding to each char sample.

### CO_2_ adsorption test

The adsorption capacity of the char and AC samples was determined using thermogravimetric analysis (TGA) in a Netzsch STA 449 Jupiter thermobalance following the procedure proposed by Gil-Lalaguna et al. (2022a). The adsorption tests were performed at 298 K (25 °C) and atmospheric pressure. For the adsorption measurements, a degasification process was performed to eliminate any possible physisorbed CO2 and moisture; for this process, approximately 80 mg of sample was heated at 150 °C and a heating rate of 10 °C/min in a N_2_ atmosphere for 1 h. Then, the temperature was lowered to 25 °C using a cooling rate of – 10 °C/min. When the reactor reached this temperature, the sample was exposed to various CO_2_/N_2_ mixtures, with a CO_2_ volume fraction ranging from 5 to 83%. A 20 mL/min STP of N_2_ was used as a protective gas flow in the TGA apparatus. The samples were exposed to each concentration of CO_2_ for 1 h to allow equilibrium between the gas phase and the adsorbent. Each experiment had a duration of 10 h and was performed in duplicate, considering the long adsorption times; for each sample, a total process of 20 h was employed. The CO_2_ adsorption capacity (mg CO_2_/g char) was calculated from the mass gain of the sample during exposure to different CO_2_ concentrations using Eq. (1):

1 $${Q}_{\mathrm{adsorbed}}=\frac{m_{\mathrm{f},\mathrm{conc}}-{m}_{\mathrm{desg}}}{m_{\mathrm{desg}}}\ast 1000\kern2.25em \left[\frac{mg_{{\mathrm{CO}}_2}}{g_{\mathrm{solid}}}\right]$$

where

*Q*_adsorbed_: quantity of CO_2_ adsorbed at different concentrations.

*m*_f,conc_: final mass of the solid at each concentration.

*m*_desg_: mass of the solid after the degasification process (considered initial mass of the solid before the adsorption process).

Once adsorption tests were performed, CO_2_ flow was replaced by pure N_2_ and the temperature in the reactor was increased to 150 °C; this led to CO_2_ desorption in case the adsorption process is reversible. Mass loss occurring when the temperature is increased indicates the reversibility of the adsorption process.

### Statistical analysis

Analysis of variance (ANOVA) was performed to compare the variation of CO_2_ adsorption performance between the biochar and activated carbons obtained from the different treatments (from torrefied and non-torrefied palm kernel shell). Fisher’s least significant difference (LSD) was performed to identify the samples whose means were statistically different. On the other hand, Student’s *t*-test for the difference of dependent means was performed to analyze the paired effects between biochar and activated carbons. This was done with the aim to identify the changes between the biochars and its subsequent activated carbons. The software Statgraphics® was used for the statistical analysis.

## Results

To obtain an efficient carbonaceous material (biochar or activated carbon) with the capability to adsorb CO_2_, two distinct routes were evaluated. The first follows the conventional approach commonly reported in the literature (pyrolysis–activation) (Li et al. 2008; Pallarés et al. 2018; Abuelnoor et al. 2021). The second route involves the experimental strategy proposed in this study, incorporating an additional torrefaction step before the pyrolysis process (torrefaction–pyrolysis–activation). Characterization of the obtained biochar from each route was conducted before activation using elemental analysis, thermogravimetric analysis, surface area analysis, and FTIR. The resulting activated carbon was characterized by surface area and SEM analyses. An additional route was implemented, involving direct activation of biomass without preceding thermal processes (torrefaction, pyrolysis), aiming to compare the impact of thermochemical conversion of biomass before the activated carbon production process.

### Elemental analysis

**Fig. 1:**
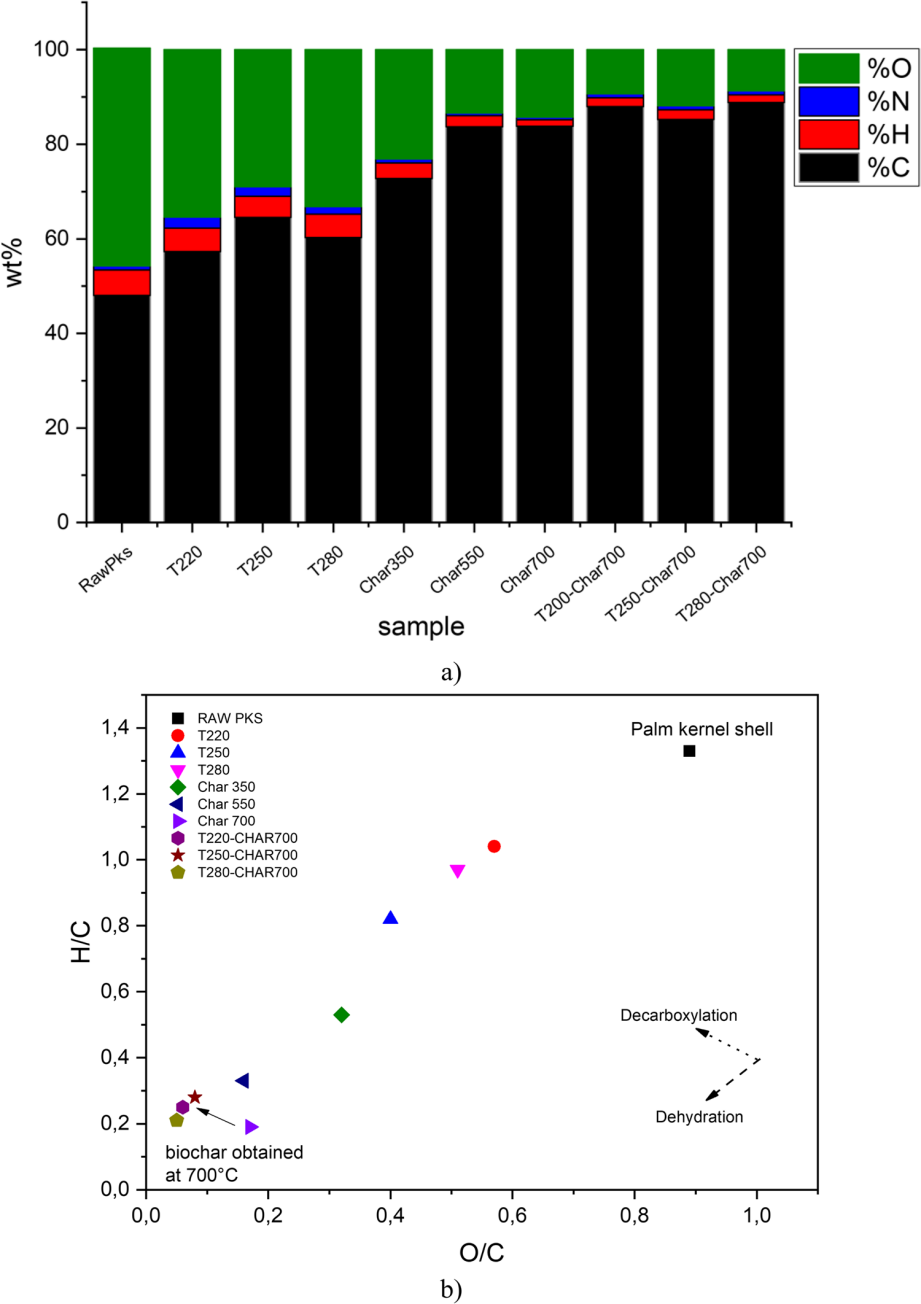
**a** Elemental analysis of biochar samples obtained from raw and torrefied palm kernel shell. **b** Van Krevelen diagram

The change in the elemental composition of the biochar samples obtained from palm kernel slow pyrolysis is presented in Fig. 1a. The raw material was initially composed of 48.08% C, 43.01% O, 5.36% H, and 0.87% N, with the sulfur content being undetectable. Carbon (C), oxygen (O), and hydrogen (H) are the major elemental constituents of biomass, and their ratios determine its fuel properties (Aninda Dhar et al. 2022). Higher O content leads to higher reactivity of biomass during thermochemical conversion (Pérez et al. 2019), in contrast to hydrogen content, which is directly related to the energy content of biomass. On the other hand, a high carbon content of biomass leads to more char formation after the pyrolysis process (Vega et al. 2019). The presence of sulfur in lignocellulosic materials can result in the generation of toxic compounds during pyrolysis, such as environmentally hazardous sulfides (SO_x_), which can lead to the generation of greenhouse gasses and acid rain (Aninda Dhar et al. 2022). Thus, palm kernel makes it a promising and environmentally friendly raw material to produce carbonaceous materials by pyrolysis. The data indicate a gradual increase in the carbon content in the biochar from 57.29 to 83.83% as the process temperature is increased from 220 to 700 °C because of the high degree of carbonization due to aromatization processes. Likewise, there is a gradual decrease in the oxygen content (43.01% to 8.31%) and the hydrogen content (5.36% to 1.34%) due to the devolatilization processes at high temperatures and the breaking of weak bonds in the solid structure (Al-Wabel et al. 2013). The nitrogen content shows a slight increase up to 280 °C (typical torrefaction range) of approximately 0.87%–2.04%.

Previous reports (da Silva Veiga et al. 2020) indicate that this behavior is due to the incorporation of nitrogen in more complex and heat-resistant structures that are unable to devolatilize easily with an increase in pyrolysis temperature. On the other hand, the ash content increases slightly in all biochar samples as the temperature increases because of the presence of inorganic components (carbonates, silicates, sulfates, phosphates) and nutrients (Na, Mg, K, Ca) that do not devolatilize during pyrolysis and, consequently, remain in the biochar structure (da Silva Veiga et al. 2020).

On the other hand, the effect of previous torrefaction is also observed. Data indicate that the previous torrefaction did not generate significant changes in the elemental composition of the biochar, increasing by 5% (83.83% to 88.85%) to more severe torrefaction and slight decreases in the oxygen content (8.32% to 4.27%) directly related to the release of oxygenated compounds during the previous torrefaction. In contrast, the hydrogen content slightly increased, going from 1.34% in non-torrefied sample to 2.03% in the previously torrefied sample at 250 °C. This behavior was confirmed by the evolution of the O/C and H/C ratios plotted on the Van Krevelen diagram of the biochar samples shown in Fig. 1b.

A decrease in the O/C (0.95 to 0.17) and H/C (1.33 to 0.19) ratios was observed as the slow pyrolysis temperature increased. This confirms the loss of oxygenated and hydrogenated compounds during the process. This behavior allows a gradual increase in the aromaticity of the obtained biochar samples, thus highlighting the importance of dehydration reactions (loss of H and O in the form of H_2_O), decarboxylation (loss of oxygenated compounds in the form of CO andCO_2_), and demethylation (loss of H in the form of CH_3_) (Suliman et al. 2016). The atomic ratios of the obtained biochar samples exhibit an interesting behavior: a decrease in the O/C ratio (0.10 to 0.05) in the previously torrefied samples, contrary to the increase in the H/C ratio (0.19 to 0.28) as the previous torrefaction temperature increases. There was a linear correlation between the changes in the O/C and H/C atomic ratios, presenting a slope $\left({~}^{{\Delta }_{H/C}}\!\left/ \!{~}_{{\Delta }_{O/C}}\right.\right)$ of 0.99. This value indicates that the release of compounds such as CO_2_ and CO derived from decarbonylation and decarboxylation reactions is dominant, and consequently, the release of oxygenated compounds is faster through these routes than through pure dehydration reactions. This behavior is attributable to the degradation suffered by hemicellulose and cellulose at these temperatures (Chen et al. 2018). Furthermore, despite its high thermal stability, the lignin fraction undergoes certain changes during torrefaction. Previous studies (Dai et al. 2018a) have reported that during the torrefaction of lignin, the release of CH_4_ and CH_3_OH through demethoxylation reactions is common, thus causing some degradation of oxygenated and hydrogenated compounds. The content of C–C and C–H bonds in the lignin structure can be favored as the release reactions of oxygenated compounds are stronger. Ben and Ragauskas (2012) attributed this behavior to several factors, including the cleavage of lignin ether bonds during the thermal process, which could re-condense in the form of aromatic C–C bonds and the conversion of some carbohydrates to aromatic C–C and C–H bonds.

### Thermal stability of the samples during pyrolysis

**Fig. 2:**
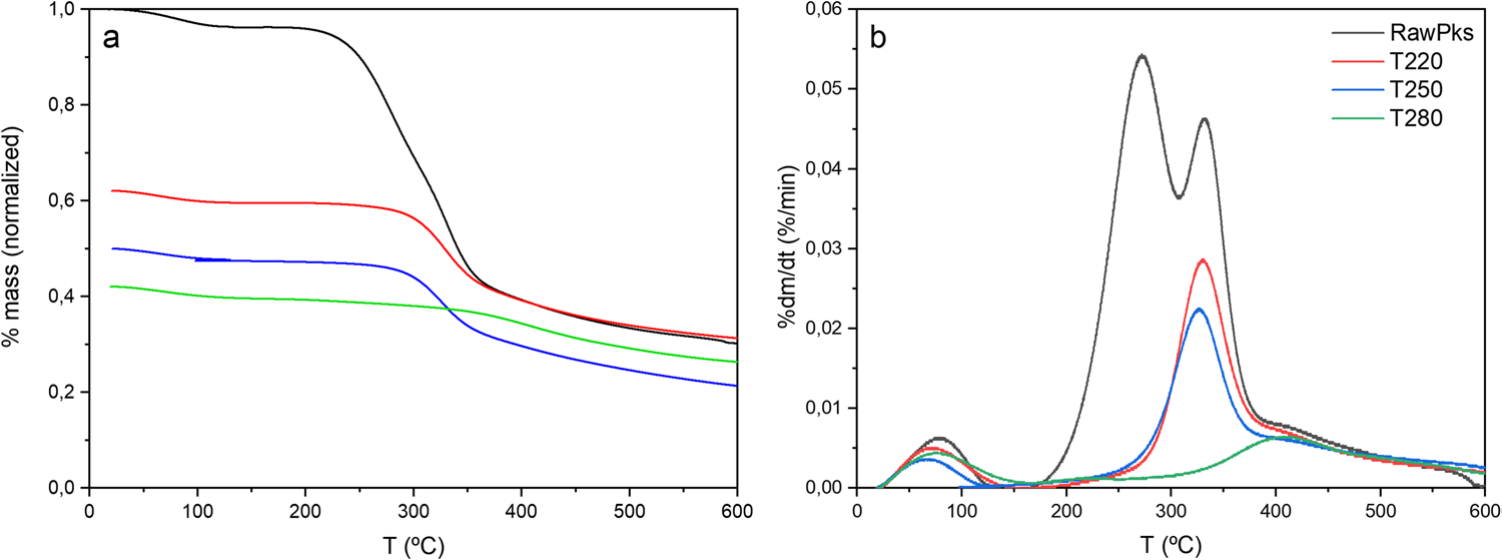
Results of thermogravimetric analysis of raw and torrefied palm kernel shell. **A** Mass loss curve (TG) and **B** DTG curve

The thermal stability of the torrefied palm kernel was measured by thermogravimetric analysis (TGA). Figure 2 shows the TG and DTG curves corresponding to the virgin and torrefied palm kernel subjected to slow pyrolysis at 700 °C and a heating rate of 10 °C/min. Three zones can be identified during the palm kernel heating process: a region between 100 and 105 °C, where residual moisture is eliminated from the sample and some extractives. Two characteristic peaks occur in the range between 190 and 280 °C and 310–380 °C, where the greatest mass loss occurs due to the release of a large part of the volatiles contained in the biomass. This can be attributed mainly to the breakdown of hemicellulose and cellulose, respectively (Ma et al. 2015). Around 400 °C, there is a shoulder with a slower loss of mass, mainly attributed to the slow degradation of lignin, more severe carbonization process, and decomposition of high molecular weight volatiles (Ma et al. 2017). Figure 2a shows the TG curve obtained from virgin and torrefied palm kernel subjected to slow pyrolysis. As a result, the mass changes during the pyrolysis of the torrefied biochar present significant differences compared to not torrefied kernel. In the torrefied palm kernel shell, the weight loss exhibited a different behavior compared with the raw palm kernel shell, demonstrating enhanced thermal stability as a product of the torrefaction process. This phenomenon can be explained by the poor thermal stability of some functional groups of hemicellulose, such as hydroxyls and side chains, and sugars related to the cellulose structure, which are sensitive to high temperatures (Wang et al. 2016, 2020). During this process, it is possible to increase the solid yield at higher torrefaction temperatures because of the enrichment of lignin in the biomass, which is attributed to the increase in the content of insoluble lignin and some alkali metals (Pelaez-Samaniego et al. 2014; Zheng et al. 2015; Cao et al. 2016).

Figure 2b shows the disappearance of the first peak in the DTG curve in samples subjected to prior torrefaction, confirming that hemicellulose is the main compound affected by thermal degradation. This behavior agrees with previous studies (Barontini et al. 2021), which reported that this peak decreases in intensity at light torrefaction temperatures and completely disappears at more severe temperatures. As expected, the second decomposition peak is gradually reduced as the torrefaction temperature increases because of the partial degradation of hemicellulose, which is higher at high temperatures (close to 30 °C). The third shoulder-shaped peak is associated with the low decomposition of lignin at these temperatures. At more severe torrefaction temperatures, only the lignin content was partially affected; thus, the complete degradation of hemicellulose and cellulose in the samples was confirmed. As the torrefaction temperature increases, hydrogen bonds in the cellulose structure of biomass are broken and the structure becomes more complicated; additionally, as the temperature increases, benzene ring units of lignin are further polymerized, increasing the aromaticity of torrefied biomass (Khairy et al. 2023).

### Analysis of the porous structure

#### Porous structure of biochar

**Fig. 3:**
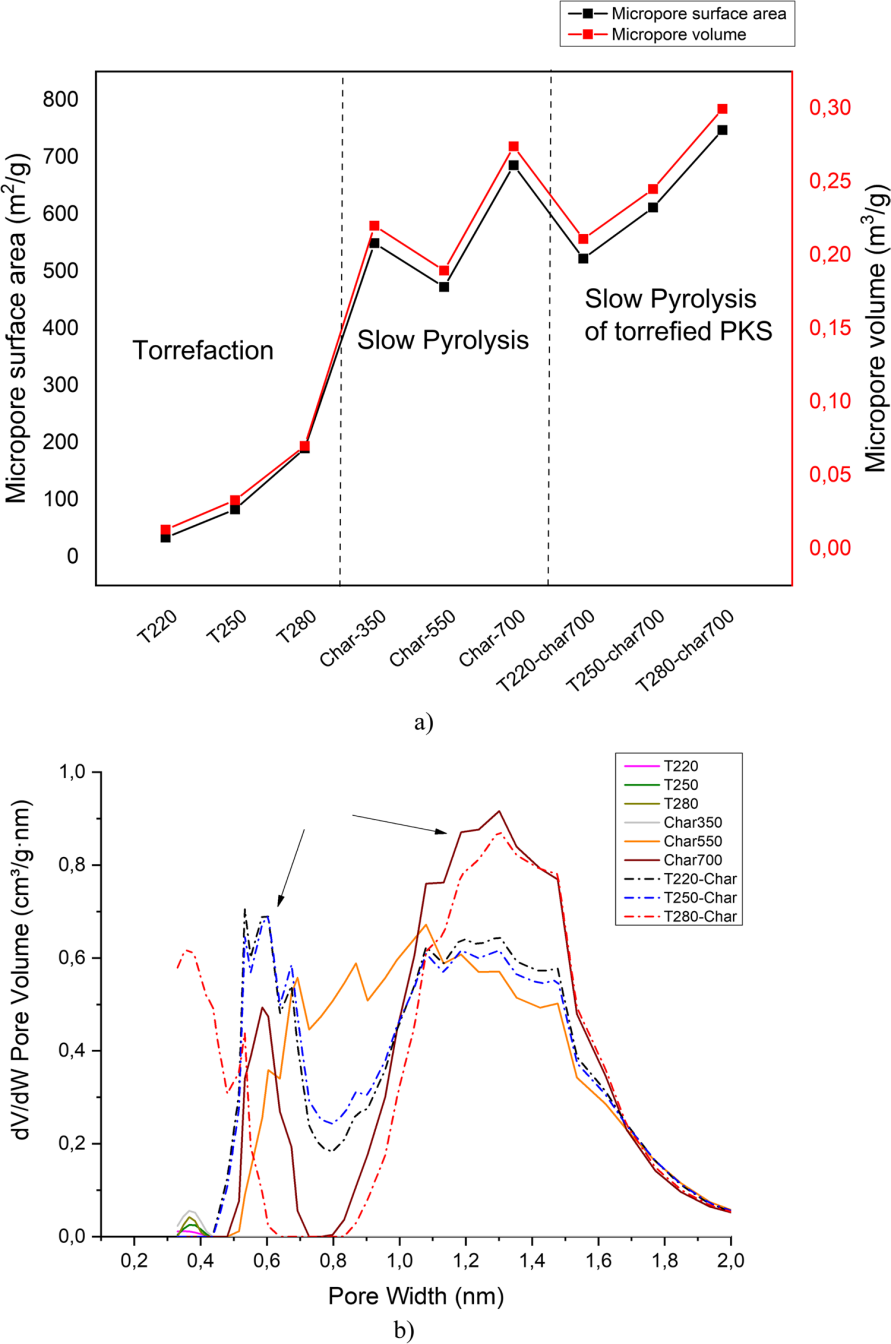
**a** Changes in micropore surface area (black lines) and micropore volume (red lines) measured with CO_2_ at 273 K of biochar obtained from raw and torrefied palm kernel shell. **b** Pore size distribution (microporous zone) measured with CO_2_ at 273 K of biochar obtained from raw and torrefied palm kernel shell

Textural parameters such as specific surface area, pore volume, and pore size distribution are determinants for evaluating the porosity of solid materials such as biochar and activated carbon. For this purpose, N_2_ and CO_2_ adsorption tests were performed on the obtained samples. Figure 3a shows the changes in the micropore surface area and micropore volume of the biochar samples obtained at temperatures between 220 and 700 °C. For the biochar samples, characterization by N_2_ adsorption was not performed because of the excessively long equilibration times required to start the measurements. This is typical of samples that exhibit high microporosity and have micropores smaller than 1 nm (Suliman et al. 2016). During the first stage (220–280 °C), in which torrefaction typically occurs, the microporous area slowly increases to 250 °C because the palm kernel volatiles begin to be released, leading to the production of new porous structures. When the pyrolysis temperature is increased to 350 °C, there is a considerable increase in the area and volume of the micropores. During this stage, dehydration, depolymerization, glycoside cleavage, decarboxylation, and de-branching reactions of cellulose and hemicellulose structures are performed. Likewise, by the continuous degradation of cellulose together with lignin to a lesser extent and the gas-solid interactions during devolatilization, the formation of more fused aromatic structures joined with one or two aromatic rings is promoted. These bonds contain methyl, methylene, and oxygen functional groups (Thommes et al. 2015; Yang et al. 2016). At 550 °C, a slight decrease in the surface area and micropore volume was observed. This can be attributed to the sintering and softening reactions of high-molecular-weight volatiles that result in a blockage of pores due to depolymerization of the molten volatiles (Jia and Lua 2008b). At these temperatures, some divisions occur in the methyl, methylene, and oxygen functional groups, resulting in a greater opportunity to be in contact with the fused aromatic rings at lower temperatures. Then, they go from having aromatic structures of one or two rings to aromatic structures of three to five rings more ordered (Yang et al. 2016). When the pyrolysis temperature is raised to 700 °C, the char reaches the maximum value of surface area and volume of micropores, respectively (685.88 m^2^/g and 0.274 m^3^/g), owing to the release of the high molecular weight volatiles by the high temperatures, as well as the destruction of certain aliphatic groups such as alkyl and ether groups. Moreover, the exposure of more groups of aromatic rings bound to lignin at these temperatures promotes a considerable increase in the specific surface area (Tomczyk et al. 2020).

**Fig. 4:**
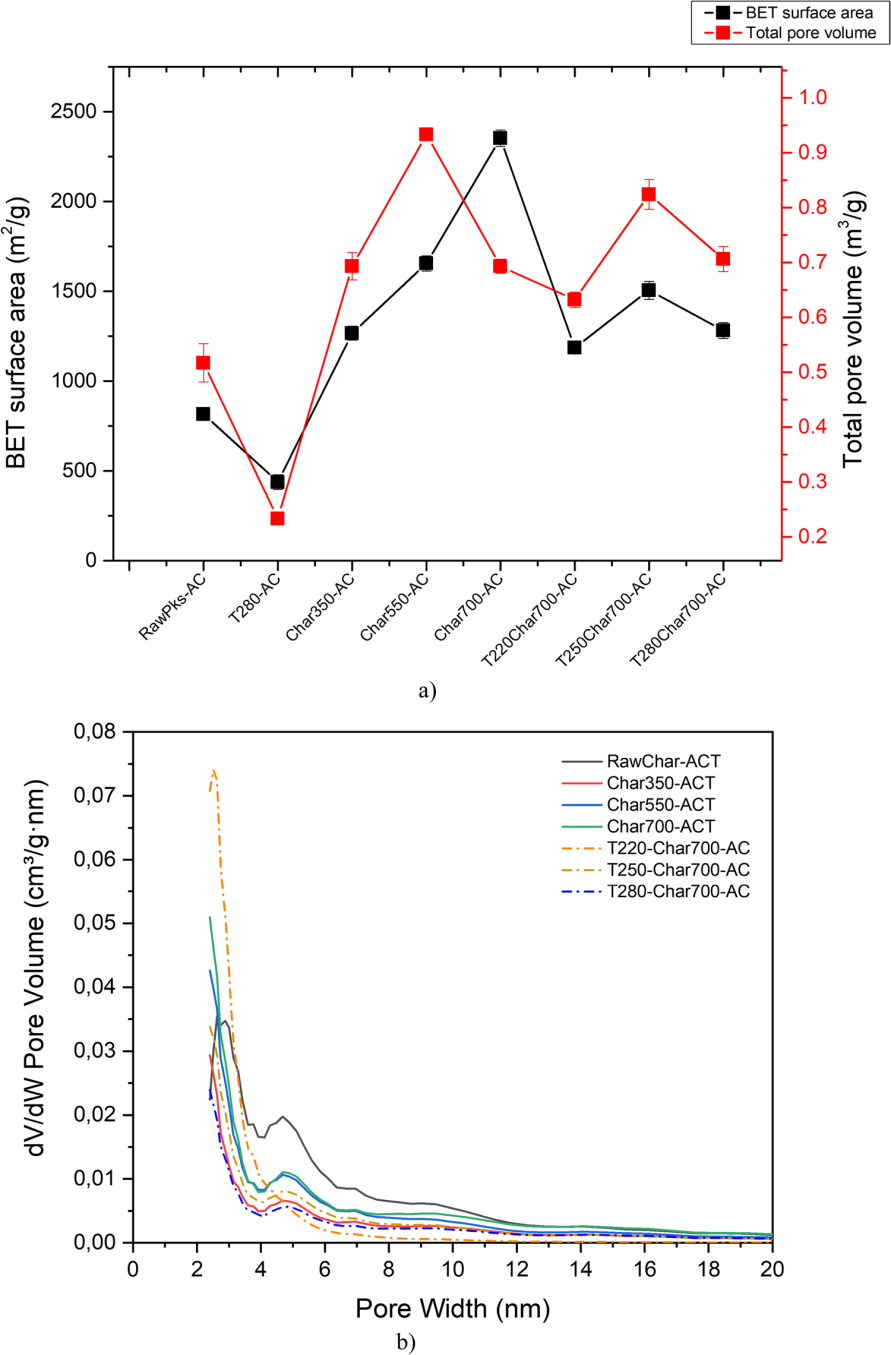
**a** BET surface area (red bars) and total pore volume (blue bars) measured with N_2_ at 77 K of activated carbons obtained from raw and torrefied palm kernel shell biochar. **b** Pore size distribution (2 – 20 nm) measured with N_2_ at 77 K of activated carbons obtained from raw and torrefied palm kernel shell’s biochar

The effect of previous torrefaction on the textural parameters of the biochar samples was also observed. The results indicate that torrefaction at low temperatures (up to 250 °C) decreases the surface area and volume of micropores in the biochar samples; likewise, at more severe temperatures, values similar to those obtained for the palm kernel without prior torrefaction are achieved. Some authors (Zhang et al. 2016) have indicated that as the pre-torrefaction temperature increases, the surface area and pore volume decrease because there is a lower number of volatiles in the structure of the torrefied solid and a lower release of these during pyrolysis. Therefore, a lower pore opening. Crosslinking reactions during torrefaction are responsible for changes in the internal structure of the biochar. In contrast, Fig. 4 shows that the development of surface area and volume of micropores is favored as the volatile material content decreases and the fixed carbon content in the biochar sample increases. This behavior confirms the changes suffered in the devolatilization of the previously torrefied palm kernel, resulting in the exposure of non-degraded compounds that delay the formation of fixed carbon in the char structure.

Figure 3b presents the pore size distribution of the biochar obtained from raw and torrefied palm kernel shell. It is possible to identify two distribution peaks in each sample: the first peak shows pores less than 0.8 nm, and the second peak shows pores between 1.2 and 1.4 nm. It evidences the marked microporosity of the material obtained. The sample obtained from the torrefied char at 280 °C shows a much higher volume than the torrefied samples at a lower temperature in the area between 1.2 and 1.4 nm. The opposite happens in the area comprising pores less than 0.8 nm. Likewise, smaller pores are formed on the order of 0.4 nm (ultra-micropores) in the same sample. This behavior confirms that the complete devolatilization of the biochar structure promotes the release of micropores and, consequently, an increase in the specific surface area.

#### Porous structure of activated carbon

**Fig. 5:**
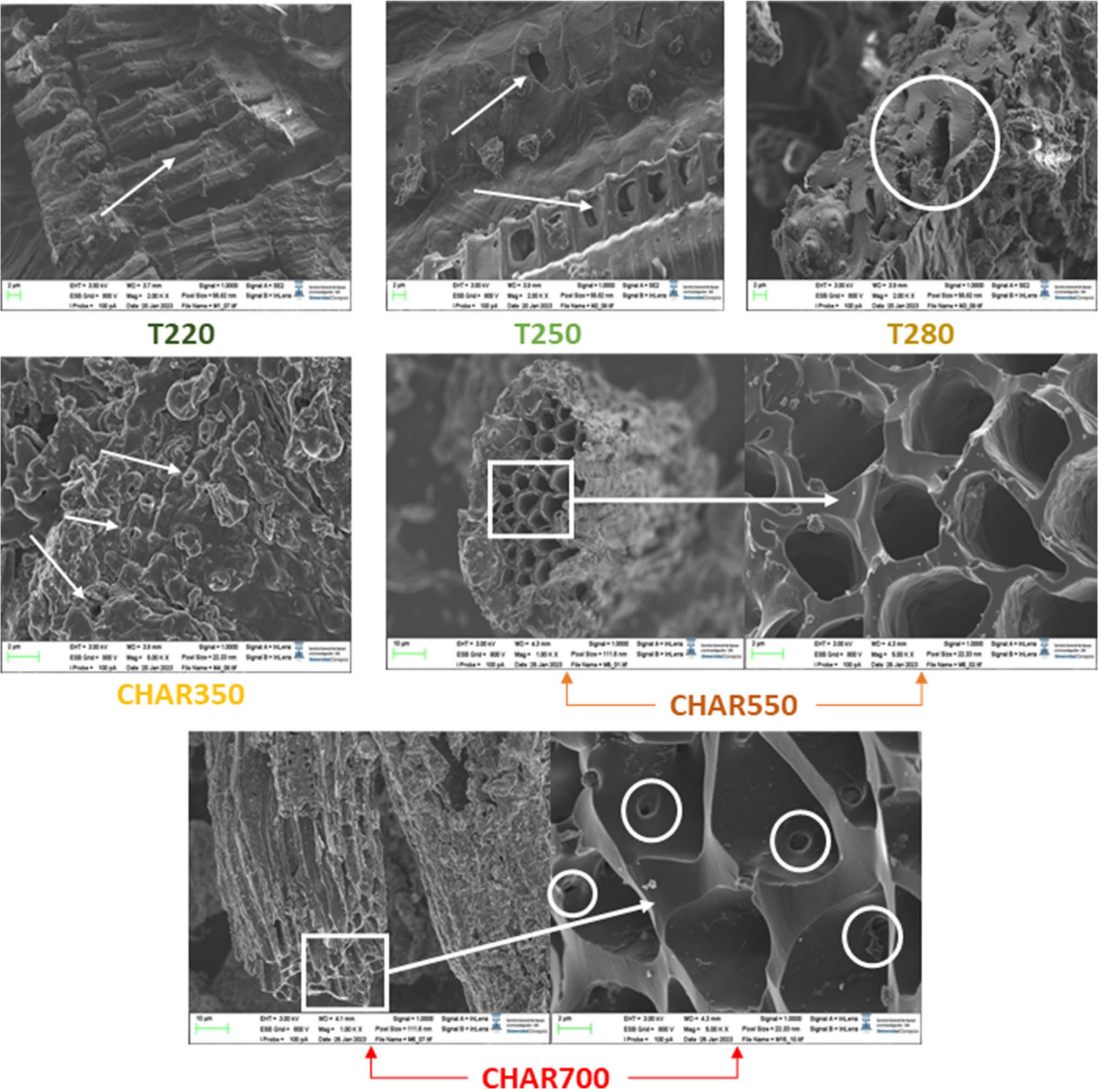
Morphological changes in biochar obtained from palm kernel shell at different torrefaction and pyrolysis temperatures

**Fig. 6:**
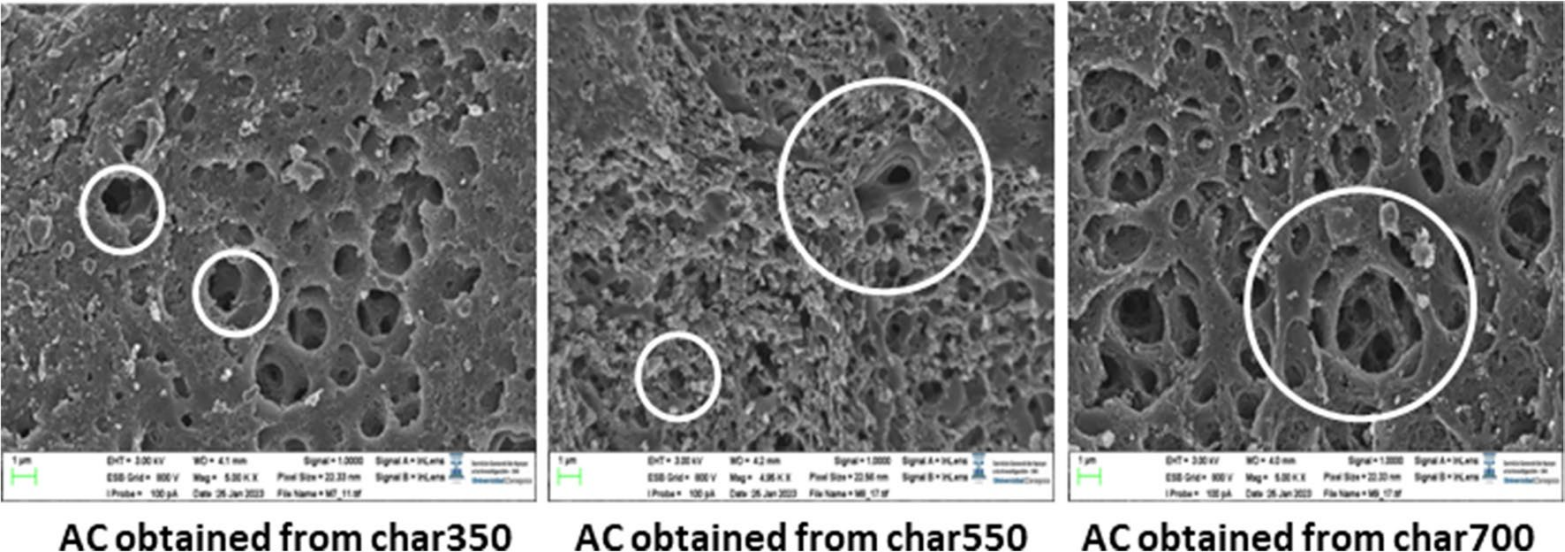
SEM micrographs of activated carbon obtained from Char350, Char550, and Char700 samples

Subsequently, activated carbon was obtained from the biochar samples. To evaluate the effect of slow pyrolysis temperature on the final characteristics of the activated carbons, the samples with the best textural properties during slow pyrolysis (Char350, Char550, and Char700) were selected. Their textural properties were evaluated using N_2_ adsorption isotherms at 77 K and CO_2_ at 273 K. Figure 6a shows the adsorption isotherms of activated carbon obtained from palm kernel and virgin palm kernel slow pyrolysis biochar. The activated carbons obtained exhibit a mixture of type I and type IV adsorption isotherms, typical of materials with micropores and mesopores. This can be confirmed in Fig. 6b from the pore size distribution of the obtained activated carbons, which ranges between 2 and 6 nm. It is possible to highlight the presence of mesopores with a greater volume for the sample obtained without prior pyrolysis. Moreover, as the material is subjected to consecutive pyrolysis, there is a progressive decrease on the peaks from 2 nm, and the micropores tend to increase. This agrees with Fig. 5a and b and demonstrates that the continuous release of volatile material during the palm kernel pyrolysis process favors the release of previously plugged micropores, which are subsequently opened by the gasifying agent. In turn, we found that an increase in the slow pyrolysis temperature of the palm kernel up to 700 °C resulted in a significant increase in the BET surface area and total pore volume of the resulting activated carbon (2353 m^2^/g and 1.291 m^3^/g, respectively). This is a result of the formation of more complete carbonaceous structures and the total release of the high- and low-molecular-weight volatile material contained in the solid structure. The latter is a product of the high pyrolysis temperatures reflected in the formation of an initial porous structure (Bouchelta et al. 2012).

On the other hand, the effect of previous torrefaction is presented. The activated carbon obtained from the char torrefied at 220 °C has a type I isotherm, typical of purely microporous materials; likewise, the other samples present a mixture of type I and type IV isotherms, typical of materials with micropores and mesopores, and a hysteresis typical of condensation processes during the desorption of gas in the analysis. As seen in Fig. 4b, the pore size distribution of the activated carbons changes with the previous torrefaction temperature. The activated carbon obtained from the char torrefied at 220 °C presents a distribution curve with a tendency to have smaller pores, whereas the other curves present peaks between 4 and 5 nm. This confirms the increase in mesoporosity in these materials, following the trend of total pore volume due to pore enlargement. Contrary to what was obtained in the char samples, an increase in the surface area was observed with an increase in the torrefaction temperature to 250 °C, and it decreased at higher temperatures. It has been reported that, during torrefaction, intermediate lignin liquids are formed and function as binders of solid particles, which may explain the decrease in surface area during more severe torrefaction (Pelaez-Samaniego et al. 2014). Several authors (Li et al. 2018; He et al. 2019) have found that torrefaction as a pretreatment reduces reactivity during gasification due to the degradation, polycondensation, and carbonization of most of the hemicellulose and fractions of the lignin during torrefaction, which are also due to the physical alteration that the solid undergoes during torrefaction.

### Surface morphology (SEM analysis)

To observe the morphological changes on the surface of the biochar, Fig. 5 presents the SEM micrographs of the biochar samples obtained at 220 °C, 250 °C, 280 °C, 350 °C, 550 °C, and 700 °C.

In general, physical changes in the physical structure of the biochar are evident as the process temperature increases. The images show the continuous degradation of the solid material as an effect of the continuous structural changes that occur in the biochar as the pyrolysis process moves on at higher temperatures (Yang et al. 2016). It is interesting to observe honeycomb-like structures composed of canals and nets in most of the obtained biochar samples, which are preserved at high process temperatures (550 and 700 °C). These structures consist of heterogeneous veins, lateral pits, and channels that originate from the cellular tissue structure of the precursor material (palm kernel) (Suliman et al. 2016). However, there are marked differences in two stages of the process: up to 280 °C, the structural damage to the surface of the solid is less marked; in the samples obtained at 220 °C and 250 °C, it is possible to see that a large part of the structure of the cell walls belonging to the original biomass is maintained. In addition, the appearance of pores on the surface can be observed. This may be due to the devolatilization of unstable polymers bound to hemicellulose. At 280 °C, the structural damage is more noticeable; there is a continuous appearance of new pores that are not easily observed at lower temperatures; however, part of the original structure remains unchanged. At higher temperatures (greater than 350 °C), the collapse of the biochar surface is more marked as the process progresses. At these temperatures (350–550 °C), the most severe devolatilization processes occur because of the complete degradation of the cellulose contained in the biomass, enabling the appearance of new porous structures clearly visible on the surface. At 700 °C, where higher micropore surface areas are obtained, we observe a smoother surface with the formation of new pores inside the channels. This confirms that the devolatilization and reorganization of the structure have been completed.

A significant increase in the porosity of all samples is evident in general and indicates that physical activation with CO_2_ significantly increased the porous structure (see Fig. 6). A clear effect of the previous pyrolysis can be noticed in the change in the morphology of the activated carbons’ porous structure. According to the obtained textural parameters, higher pyrolysis temperatures allow the generation of biochars with a more defined porous structure that serve as a platform for the access of CO_2_ molecules to more sites within the network. Therefore, ensuring complete devolatilization and restructuring of the solid before the activation process is relevant to the process. When the pyrolysis temperature is lower (350 °C), the resulting activated carbon maintains a slightly smoother and continuous surface and exhibits less accessible pore formation.

### FTIR analysis

**Fig. 7:**
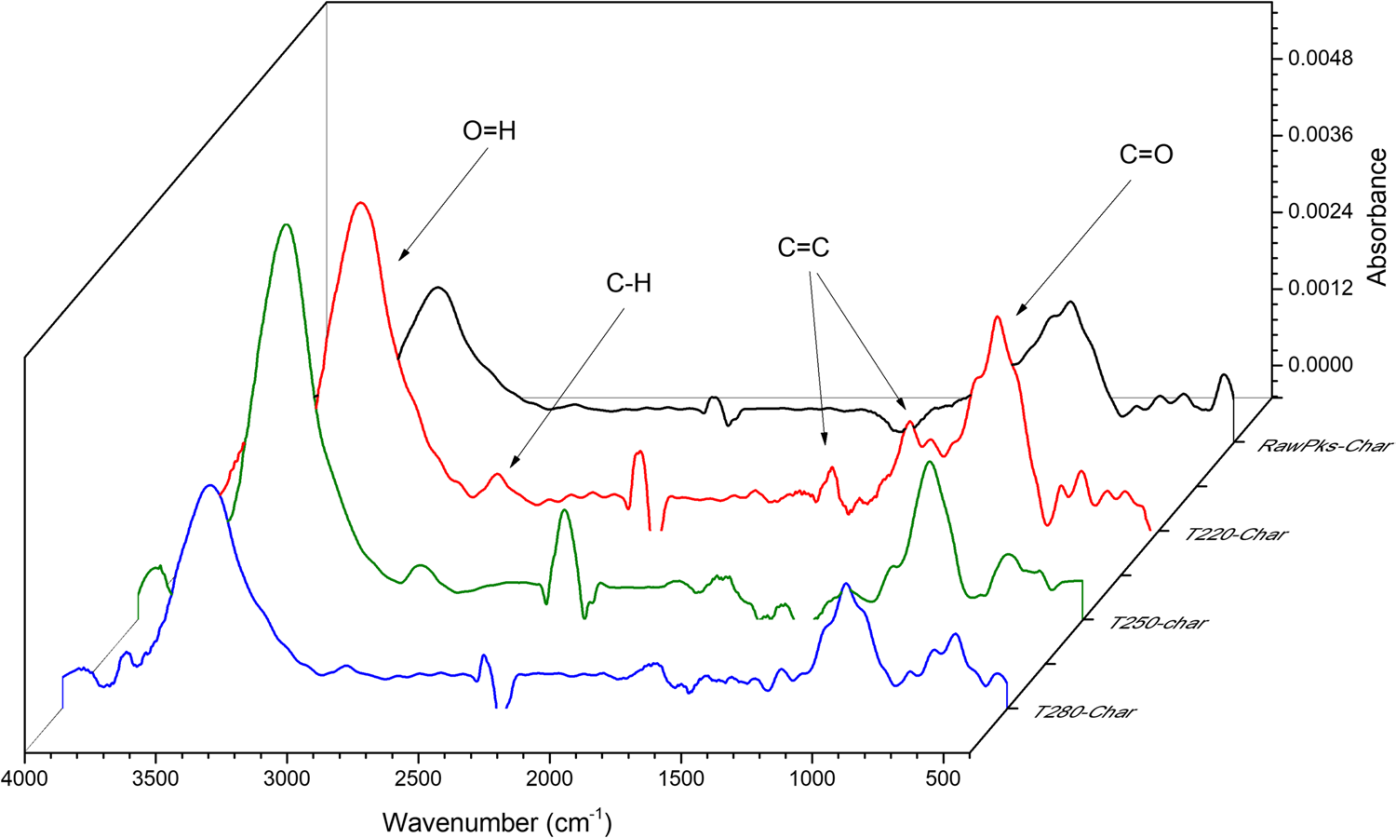
FTIR spectra of biochar obtained from raw palm kernel shell (Char700) and torrefied palm kernel shell (T220-Char700, T250-Char700, and T280-Char700)

The evolution of functional groups was evaluated by FTIR analysis and is presented in Fig. 7. It is possible to highlight five characteristic functional groups in the biochar samples: the first band—one of the most notorious—is located between the 3500 and 3300 cm^−1^ wavenumbers related to the stretching vibration of O–H groups and possibly linked to hydrogenated structures and water linked to the solid structure (Orrego Restrepo 2021). The peak located between the 2900 and 2700 cm^−1^ wavenumbers is attributed to the stretching vibrations of C–H groups coming from the aliphatic compounds –CH_2_ and alkanes-CH_3_ (Aninda Dhar et al. 2022). A weak peak occurs at wave number 2130 cm^−1^ and is attributed to C=O groups related to the CO_2_ released during pyrolysis (Orrego Restrepo 2021). The peak located between 1600 and 1400 cm^−1^ is attributed to the stretching vibration of C=C and C=O groups corresponding to aromatic groups derived from lignin compounds. Likewise, a peak between 1200 and 1000 cm^−1^ caused by the stretching vibration of C–O groups derived from phenols (Ma et al. 2017) and polysaccharides located on the cell wall of biomass.

In general, as the pyrolysis temperature increases, the intensity of the functional groups decreases and –OH, phenolic groups, and—to a lesser extent—some aliphatic and aromatic compounds prevail. In the original biomass, most of the functional groups are weakly linked to the three main components (cellulose, hemicellulose, and lignin). Hence, when subjected to the thermal process, they are usually eliminated with those compounds, especially cellulose and hemicellulose, which undergo the greatest degradation during pyrolysis.

The changes in functional groups induced by torrefaction before pyrolysis were also evaluated. It is interesting to note the increase in the intensity of the band corresponding to the –OH groups as the torrefaction temperature increases, a trend contrary to that reported by several authors (Shang et al. 2012; Kanwal et al. 2019). They confirmed that as the severity of the torrefaction increases, mainly carboxylic groups associated with hemicellulose degrade and decrease these band. Previous reports (Chen et al. 2018; Dai et al. 2018b) indicate that during the degradation of hemicellulose in torrefaction, the deoxygenation reactions are faster and prevail over the dehydration reactions. On the other hand, the intensity of the peak at 1200 cm^−1^ decreases with increasing torrefaction temperature, indicating the effect of deoxygenation and degradation of cell wall polysaccharides. The C=C functional groups related to aromatic compounds derived from lignin show a slight decrease with increasing torrefaction temperature, indicating partial degradation of lignin due to its high thermal stability. According to Khairy et al. (2023) decarbonylation and decarboxylation promote the reduction of C=O functional groups; additionally, as the torrefaction temperature increases, the reduction of C–C absorption bands can occur due to lignin heat breakdown and the formation of phenylpropane structural units of lignin in the torrefaction range of temperatures.

This can be confirmed with the results obtained in the elemental analysis (see Fig. 1), which show a direct correlation among the presence of the –OH groups, the increase in the hydrogen–carbon ratio, and the decrease in the oxygen–carbon ratio in the char. During the torrefaction process, carbohydrate breakdown was evident, resulting in a reduction of C=O and C–O molecular bonds and aliphatic bonds. This finding, in correlation with elemental analysis, confirms that the oxygenated groups are primarily degraded attributable to deoxidization impact of torrefaction (Khairy et al. 2023).

**Fig. 8:**
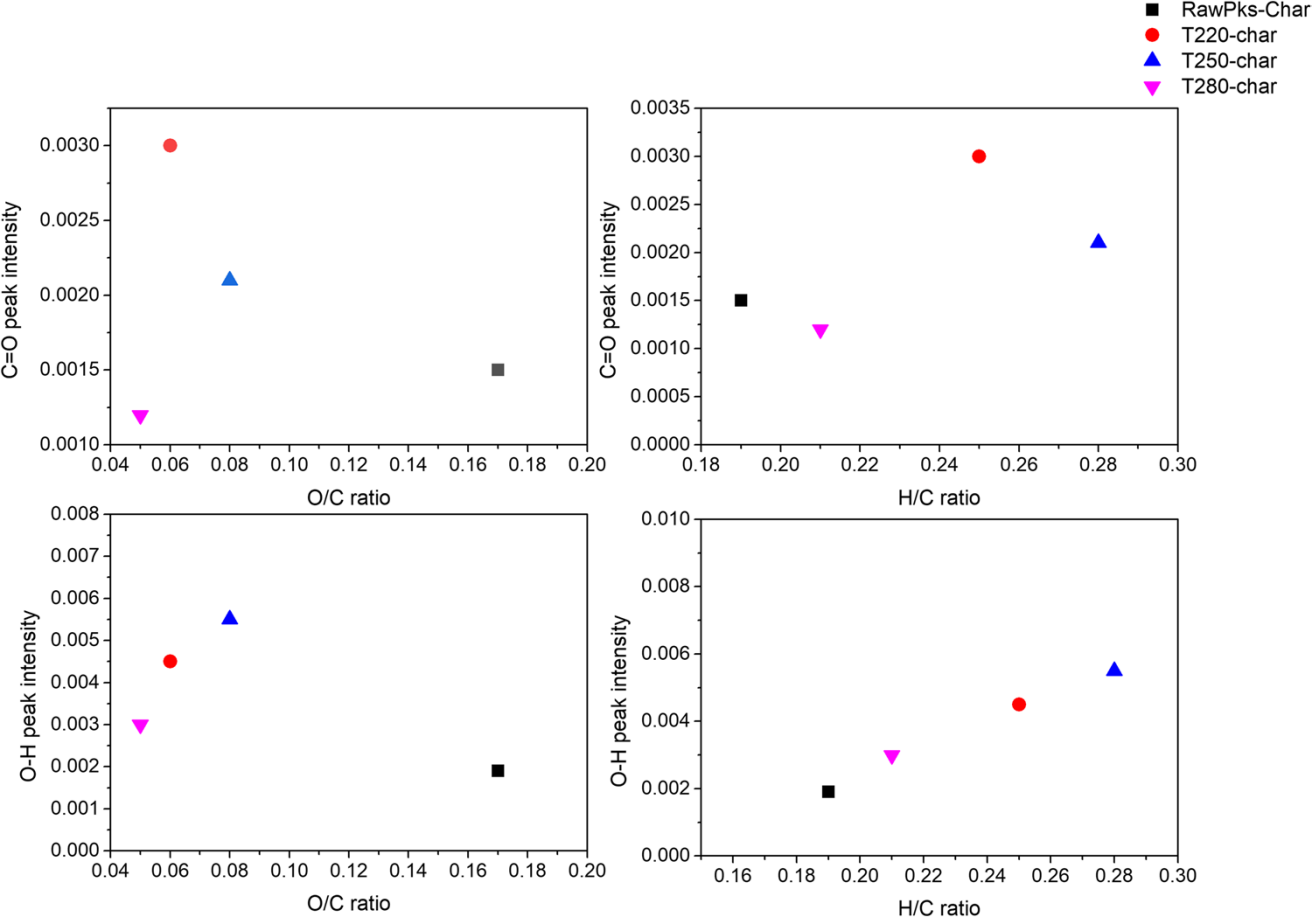
Relationship between the change in O/C and H/C ratio with peak intensity –OH (**a**, **b**) and the change in carbon and oxygen composition with C=O peak intensity (**c**, **d**)

Based on Fig. 8, it can be concluded that the increase in the OH groups and the decrease in the oxygen content and oxygen–carbon ratio in the torrefied product obtained at 280 °C are mainly due to the release of oxygenated compounds via CO and CO_2_ formation during the torrefaction process, which could influence the presence of new active sites on the char surface.

### CO_2_ adsorption performance

**Fig. 9:**
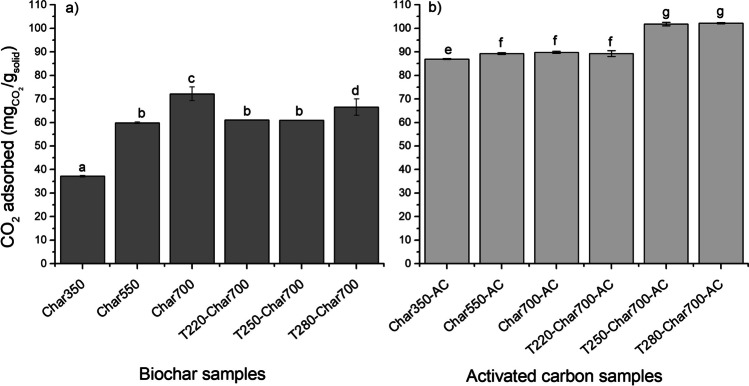
CO_2_ adsorption capacity measured at a *p*/*p*_0_ = 0.83 of (**a**) biochar obtained from raw and torrefied palm kernel shell and (**b**) activated carbon obtained from biochar samples. Columns with similar letters indicate that there are no significant differences between the means of these groups

The CO_2_ adsorption performance of the samples was evaluated using thermogravimetry at 25 °C (298 K), a temperature more representative of practical CO_2_ removal operations in gas streams, as compared to the commonly used 0 °C (273 K) for surface property measurements. Figures 8 and 9 illustrate the change in the CO_2_ adsorption capacity at a partial pressure *p*/*p*_0_ = 0.83 of biochar and activated carbon samples obtained from raw and torrefied palm kernel shell. To determine the statistical differences between CO_2_ adsorption capacity of adsorbent prepared, an ANOVA was performed for biochar and activated carbon samples independently. The ANOVA confirmed the existence of a statistical difference (*p*-value ≤ 0.05) among the samples; therefore, Fisher’s least significant difference (LSD) was performed to identify the samples whose means were statistically different. Student’s *t*-test for the difference in dependent means was performed to analyze the paired effects between biochar and activated carbons. Results presented from this analysis in Fig. 9 reveal that the samples can be classified into seven homogeneous groups with similar adsorption capacity: group A, corresponding to the Char350 sample, which has the lowest adsorption capacity (37.167 ± 0.335 mg/g); group B corresponding to the biochar obtained at 550 °C (Char 350), and biochars obtained from torrefied palm kernel shell at 220 and 250 °C (T220-Char700 and T250-Char700); group C corresponding to biochar obtained from palm kernel shell at 700 °C (Char700); group D, corresponding to biochar obtained from torrefied palm kernel shell at 280 °C (T280-Char700); group E, activated carbon obtained from the Char350 sample; group F, corresponding to activated carbons obtained from Char550, Char700, and T220-Char 700; and group G, corresponding to activated carbons obtained from T250-Char700 and T280-Char700.

From statistical analysis, significant differences between the adsorption performance of biochar and activated carbon samples can be observed. It is clear that the physically activated samples generally displayed higher CO_2_ retention capacities than the non-activated chars (increasing from 37.16 ± 0.335–72.146 ± 2.925 to 86.96 ± 0.17–102.2 ± 0.29 mg/g for activated carbons). This observation aligns with the findings from the textural properties analysis presented in Fig. 3, confirming that a well-developed porosity, featuring both micropores and mesopores, along with a high surface area, enhances the overall adsorption performance. Previous research (Gil-Lalaguna et al. 2022b) highlighted the significant role of micropores in the adsorption of CO_2_ by carbonaceous materials. The micropore-filling mechanism proposed by Dubinin (1989) suggests that high microporosity is primarily responsible for CO_2_ retention. For the char samples, it could be observed that torrefaction has little effect on the CO_2_ uptake, which is also reduced for those previously torrefied at low temperatures (220 and 250 °C with 61.036 ± 0.045 and 60.93 ± 0.05 mg/g, respectively). This behavior has a similar trend with the evolution of the microporous surface (see Fig. 3); samples with the highest microporous surface have the highest CO_2_ adsorption capacity; this can be confirmed with the pore size distribution (Fig. 3b), where a peak is observed at pore sizes < 1 nm. These results suggest that micropore filling is the main mechanism for CO_2_ adsorption in char samples (Gil-Lalaguna et al. 2022b) and can be contrasted with Table S2, which shows that a multilayer adsorption mechanism is observed for these samples, considering that the Freundlich model has the best fit with the experimental results obtained on CO_2_ adsorption isotherms at 298 K.

Adsorption isotherms presented in Fig. S1 provide further evidence supporting this behavior through comparison with pore size distribution analysis (0–2 nm) obtained from CO_2_ adsorption at 273 K using NLDFT models. Initially, the samples exhibit similar behavior at low partial pressures (lower than 0.1); in this range of concentrations, all the samples (except for sample char350) exhibit a very similar amount of CO_2_ adsorbed revealing that, at low concentrations, CO_2_ is adsorbed faster and more efficiently on the carbonaceous materials obtained regardless of their textural properties. As the CO_2_ concentration increases during the adsorption process, evident changes are observed in the behavior of each sample during the tests. The presented results clearly demonstrate that while the surface area of micropores exhibits a slight decrease after the activation process, micropores smaller than 1 nm, which are primarily involved in the adsorption process, show significant development compared with those found in the biochar samples. This, coupled with the well-developed mesopores resulting from the activation process, enhances the availability of a greater number of active sites, thereby facilitating the retention of a larger amount of CO_2_. Table S1 shows the parameters obtained from Langmuir and Freundlich adsorption models of biochar and activated carbons obtained from raw palm kernel shell. The results obtained show that there is a good correlation between the experimental data and the two models; however, Freundlich model presents the best adjustment (*R*^2^ > 0.99). In agreement with these results, a multilayer adsorption mechanism is prevalent on both biochar and activated carbon samples because of the heterogeneous surfaces of these samples (Nasri et al. 2014).

**Fig. 10:**
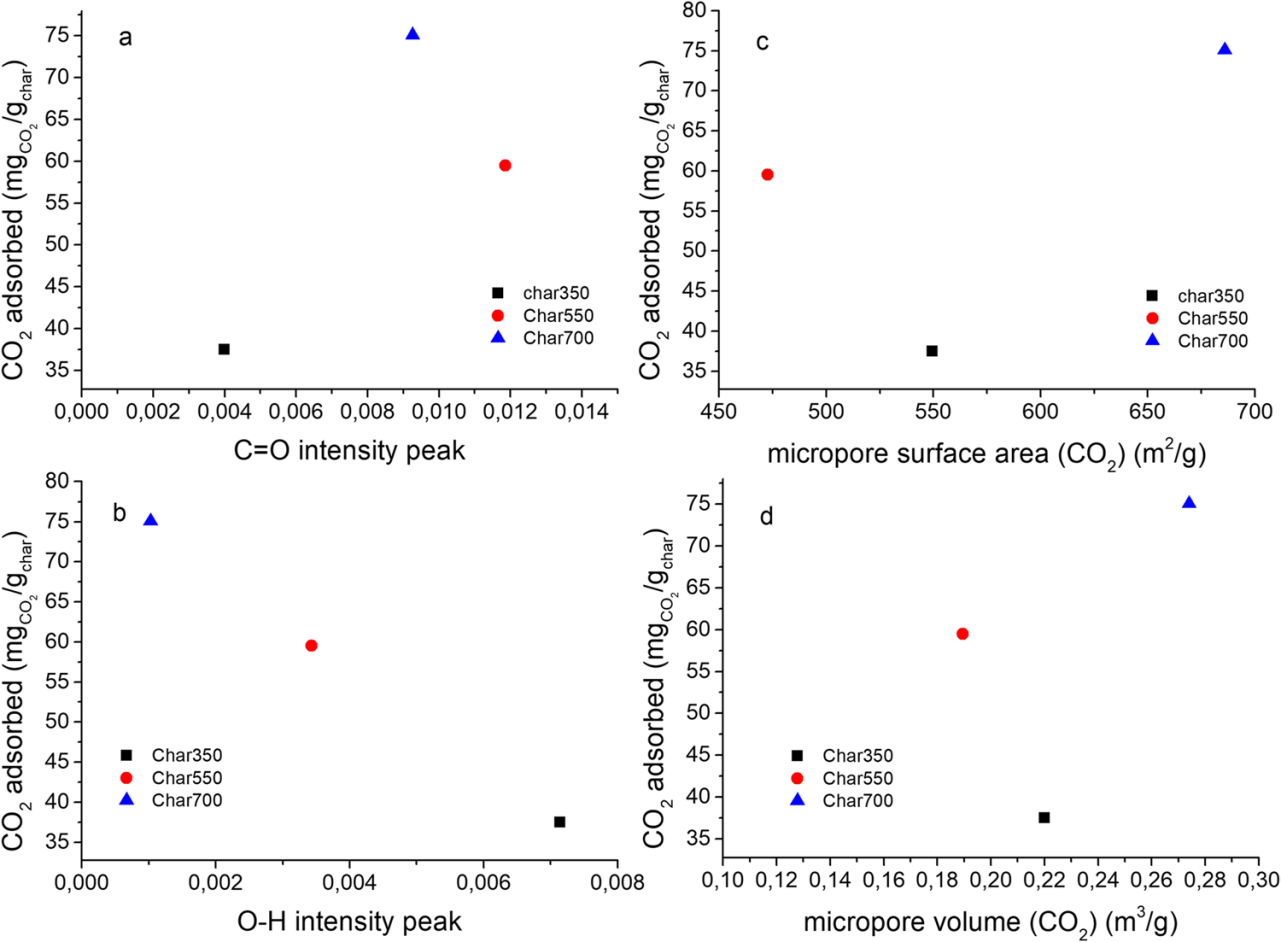
Relationship between functional groups and textural properties of char samples obtained at different pyrolysis temperatures and CO_2_ adsorption capacity. (**a**) C=O functional groups, (**b**) O–H functional groups, (**c**) micropore surface area and (**d**) micropore volume

A different trend was observed for the activated carbon samples; although there were no significant differences in adsorption capacity, the previously torrefied samples had a higher adsorption capacity than the char samples (102.2 mg/g for AC-T280Char700 sample). No direct correlation was observed between the adsorption capacity and the BET surface area of the activated carbons; however, the evolution of the total pore volume was similar to the CO_2_ adsorption behavior of the previously torrefied samples. Figure 10 shows the relationship between the adsorption capacity and the abundance of O–H and C=O groups, for biochar samples obtained under different slow pyrolysis temperatures. An evident reduction in CO_2_ adsorption capacity is observed as the prevalence of O–H phenolic functional groups increases, in contrast to the evident augmentation observed as the abundance of C=O carboxyl functional groups increases. This phenomenon could be explained by alterations in the surface charge associated with these groups. These oxygenated groups confer negative charges on the surface, inducing a modification in the electronegativity of the solid. Consequently, the heightened abundance of carboxyl groups on the surface enhances surface polarity because of their propensity to accept electrons from adjacent atoms. Consequently, an elevated capacity to interact with CO_2_ molecules, characterized by covalent bonds, ensues. Upon contact with the surface, these CO_2_ molecules engage in electron exchange with the C=O groups, resulting in quadrupolar momentum. This dynamic translates into enhanced adsorption capacity (Khosrowshahi et al. 2022). Previous research conducted by Chen et al. (2016) suggests that the CO_2_ adsorption capacity at 25 °C does not directly correlate with the textural properties of the activated carbon; these authors suggest that there are synergistic effects between the textural properties of the solid and its chemical composition, with better performance of those with intermediate surface areas and nitrogen content. This aligns with a previous study presented by Ding and Liu (2020), which suggest that as the presence of oxygen-containing functional groups increases, CO_2_ adsorption becomes more effective because of the presence of more active sites.

**Fig. 11:**
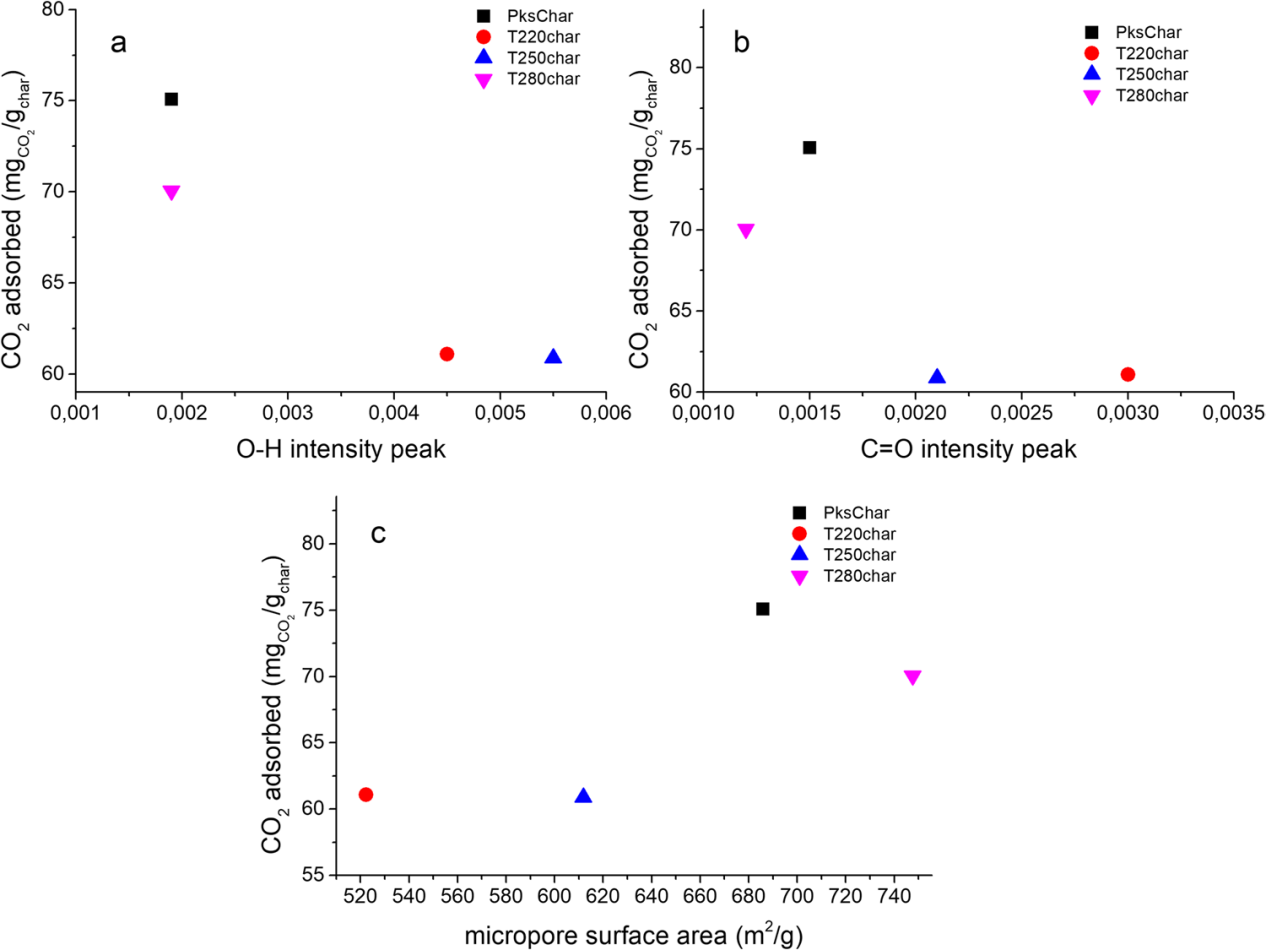
Relationship between functional groups, micropore surface area, and CO_2_ adsorption capacity of char obtained from torrefied palm kernel shell. (**a**) O–H functional groups, (**b**) C=O functional groups and (**c**) micropore surface area

A particular trend was observed for char samples obtained from torrefied palm kernel shells (Fig. 11). On the basis of the obtained results, the dominant effect of the developed surface area in the samples is confirmed, substantiating that an increase in available pores enhances CO_2_ adsorption. Conversely, a minimal influence of the oxygenated functional groups present on the surface is observed, leading to the conclusion that while the surface chemistry of the oxygenated groups indeed promotes interaction with CO_2_, the physical gas-solid interactions hold greater relevance for the retention of this gas. In contrast, Sanz-Pérez et al. (2015) found that the CO_2_ uptake capacity at 1 bar does not seem to correlate with textural properties; however, the presence of functional groups and active sites can enhance the adsorption capacity of sorbent materials. Rashidi and Yusup (2019) reported that the CO_2_ adsorption performance of activated carbons obtained from palm kernel shell is dependent on a combination of physicochemical parameters. According to this study, activated carbons with intermediate properties (carbon content, BET surface area, and pore volume) presented the best adsorption capacity, indicating synergistic effects between textural properties and elemental composition. Based on these previous studies, it can be interpreted that there are synergistic effects between the textural parameters and the physicochemical composition of the samples obtained, positively influenced in the activated carbon samples obtained, observing an improved CO_2_ adsorption capacity.

The reusability of the adsorbent is an important factor in determining the feasibility of the material in practical applications. Table 1 summarizes the CO2 uptake results obtained at *p*/*p*_o_ = 0.83 for chars and activated carbon after a second desorption–adsorption cycle. In general, a slight loss of CO_2_ adsorption capacity (less than 1%) was observed for most of the samples; Char700 and T280-Char700 exhibited a loss of adsorption capacity of 7.79% and 9.95%, respectively. These preliminary results suggest that the CO_2_ adsorption in the evaluated samples is reversible. Aljohani et al. (2023) suggested that the reversibility of an adsorbent could be reduced due to blockage of adsorption sites during each adsorption cycle. On the other hand, the structural stability of an adsorbent material can benefit the adsorption performance during repetitive adsorption–desorption cycles (Almahri et al. 2023b). However, more work is needed to confirm these results. The apparent reversibility of the process supports the idea that the contribution of physical adsorption through micropore filling plays an important role in the adsorption mechanism instead of chemical adsorption enhanced by the precipitation of CO_2_ with minerals on the solid surface (Gil-Lalaguna et al. 2022a). Further analysis is required to confirm this possibility.

**Table 1:** Performance of biochar and activated carbon samples in the cyclic adsorption–desorption test (mg CO_2_/g_char_ adsorbed at *p*/*p*_0_ = 0.83)

Sample	Cycle #1	Cycle #2
Char350	37.48	36.85
Char550	59.51	60.18
Char700	75.07	69.22
T220-Char700	61.08	60.99
T250-Char700	60.88	60.98
T280-Char700	70.05	63.08
Char350-AC	86.79	87.13
Char550-AC	89.63	88.93
Char700-AC	90.29	89.37
T220-Char700-AC	90.46	87.97
T250-Char700-AC	102.61	101.1
T280-Char700-AC	101.91	102.49

## Conclusions

This study focused on the preparation of biochar and activated carbon from torrefied palm kernel shell for CO_2_ capture applications. Experimental routes of pyrolysis-activation and torrefaction-pyrolysis-activation were conducted to explore the effects of torrefaction on the physicochemical properties of the prepared solid materials and their adsorption performance. It is crucial to highlight that the pyrolysis temperature proved to be a decisive factor in the development of micropores in the obtained biochar; the thermal processes of devolatilization and degradation of cellulose and hemicellulose-derived compounds are more pronounced at higher temperatures. On the other hand, one of the most significant findings was the pronounced influence of torrefaction on chemical reactions, particularly decarboxylation and decarbonylation, resulting in a higher release of CO and CO_2_ compounds compared with dehydration reactions. In addition, torrefaction was observed to promote an increase in the lignin content in the resulting solid, favoring the presence of C–C and C–H bonds on the solid surface. As a result, the activated carbon obtained from torrefied palm kernel shell biochar exhibits an enhanced adsorption capacity (102.2 ± 0.29 mg/g) compared with activated carbon from untreated palm kernel shell (89.83 ± 0.46 mg/g), possibly due to the emergence of new active sites interacting with CO_2_.

In summary, these results provide a profound understanding of the chemical changes that occur during torrefaction and their direct impact on the composition and porosity of biochar. These findings not only contribute to the fundamental knowledge of thermochemical processes but also offer valuable insights for practical applications, such as efficient CO_2_ capture in various industries. As future steps, a detailed study of structural stability and material reusability is suggested, along with strategies for the recovery and subsequent use of captured CO_2_. These aspects are crucial for validating the long-term viability and efficiency of these materials in practical applications.

### Supplementary information

ESM 1 (DOCX 334 kb)

## Data Availability

The datasets generated during the current study are not publicly available due to confidentiality requirements, but are available from the corresponding author on request.
