# Supplementary material for: Experimental strategy for the preparation of adsorbent materials from torrefied palm kernel shell oriented to CO2 capture
Source: Environ Sci Pollut Res Int. 2024 Feb 13;31(12):18765–84. doi: 10.1007/s11356-024-32028-3 (PMC11289003; doi:10.1007/s11356-024-32028-3)
Supplement: Supplementary file 1 — (DOCX 334 kb) [file 11356_2024_32028_MOESM1_ESM.docx]

**Supporting information**

**Experimental strategy for the preparation of adsorbent materials from torrefied palm kernel shell oriented to CO2 capture.**

*Marlon Cordoba-Ramirez^1,2*^, Farid Chejne^2^, Jader Aleán^1^, Carlos A. Gómez^2^, África Navarro-Gil^3^ Javier Abrego^3^, Gloria Gea^3^*

^1^Universidad de La Guajira, Faculty of Engineering, Mechanical Engineering Program – DESTACAR Research Group, km 3+354 via Maicao, 440001, Riohacha, Colombia.

^2^Universidad Nacional de Colombia Sede Medellín, Faculty of mines, Department of Processes and Energy – Applied Thermodynamics and Alternative Energies Research Group, Cra. 80 No 65 – 223, 050034, Medellín, Colombia

^3^Thermochemical Processes Group (GPT), Aragon Institute for Engineering Research (I3A), Universidad de Zaragoza, Edificio I+D, C/Mariano Esquillor s/n, 50018 Zaragoza, Spain.

***Corresponding Author**: Marlon Cordoba-Ramirez.

Email: [mfcordoba@uniguajira.edu.co](mailto:mfcordoba@uniguajira.edu.co)

**Figure S1.** (A) CO_2_ Adsorption isotherms measured at 298 K and atmospheric pressure of biochar and activated carbon obtained from raw palm kernel shell. (B) Micropore size distribution of biochar and activated carbons obtained from palm kernel shell.


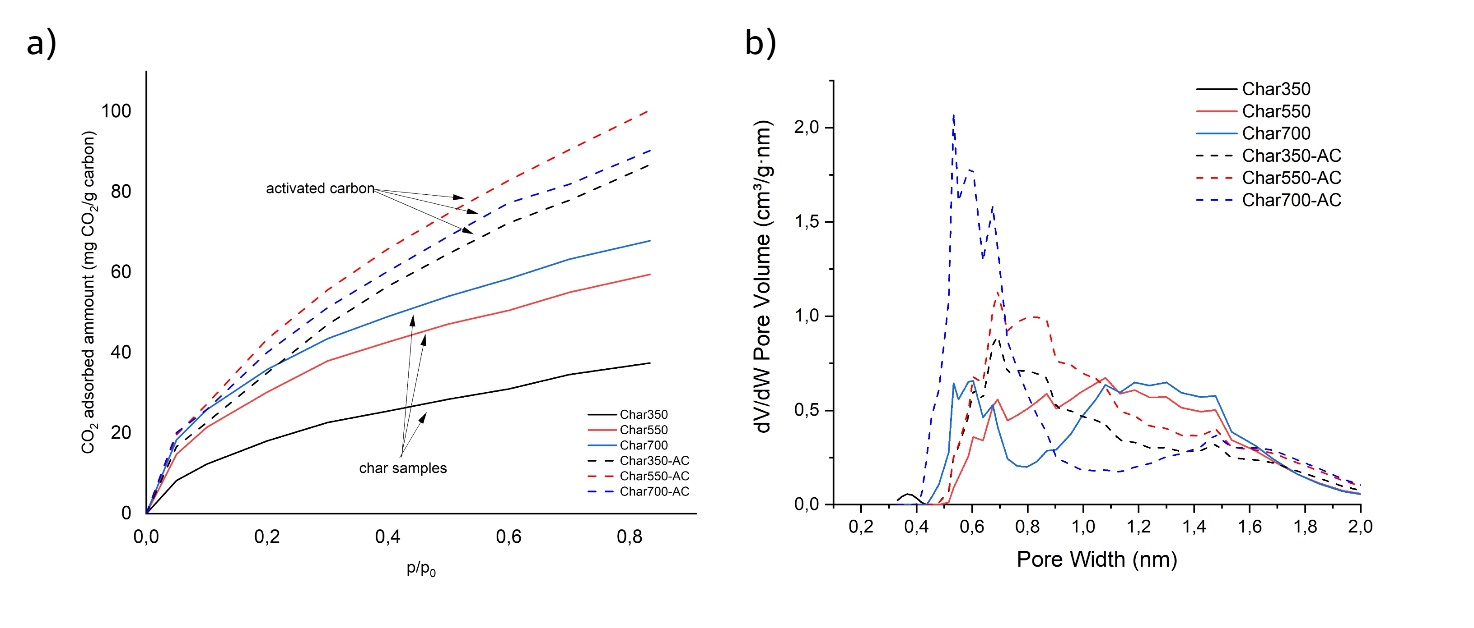


**Table S1.** Langmuir and Freundlich parameters obtained from CO_2_ adsorption isotherms at 298 K of biochar and activated carbon from raw palm kernel shell.

| **Sample** | **Langmuir** | | | **Freundlich** | | |
| --- | --- | --- | --- | --- | --- | --- |
|  | **C_m_** | **K_g_** | **R^2^** | **n** | **K_f_** | **R^2^** |
| Char350 | 39,37 | 5,08 | **0,9849** | 41,65 | 1,88 | **0,9985** |
| Char550 | 61,73 | 6,00 | **0,9814** | 66,21 | 2,03 | **0,9983** |
| Char700 | 69,44 | 6,86 | **0,9697** | 76,82 | 2,10 | **0,9962** |
| Char350-AC | 89,29 | 4,31 | **0,9574** | 97,10 | 1,65 | **0,9966** |
| Char550-AC | 105,26 | 4,32 | **0,9678** | 112,20 | 1,69 | **0,9984** |
| Char700-AC | 89,29 | 5,33 | **0,9435** | 100,14 | 1,80 | **0,9953** |

**Figure S2.** CO2 Adsorption isotherms measured at 298 K and atmospheric pressure of biochar and activated carbon obtained from torrefied palm kernel shell.

**
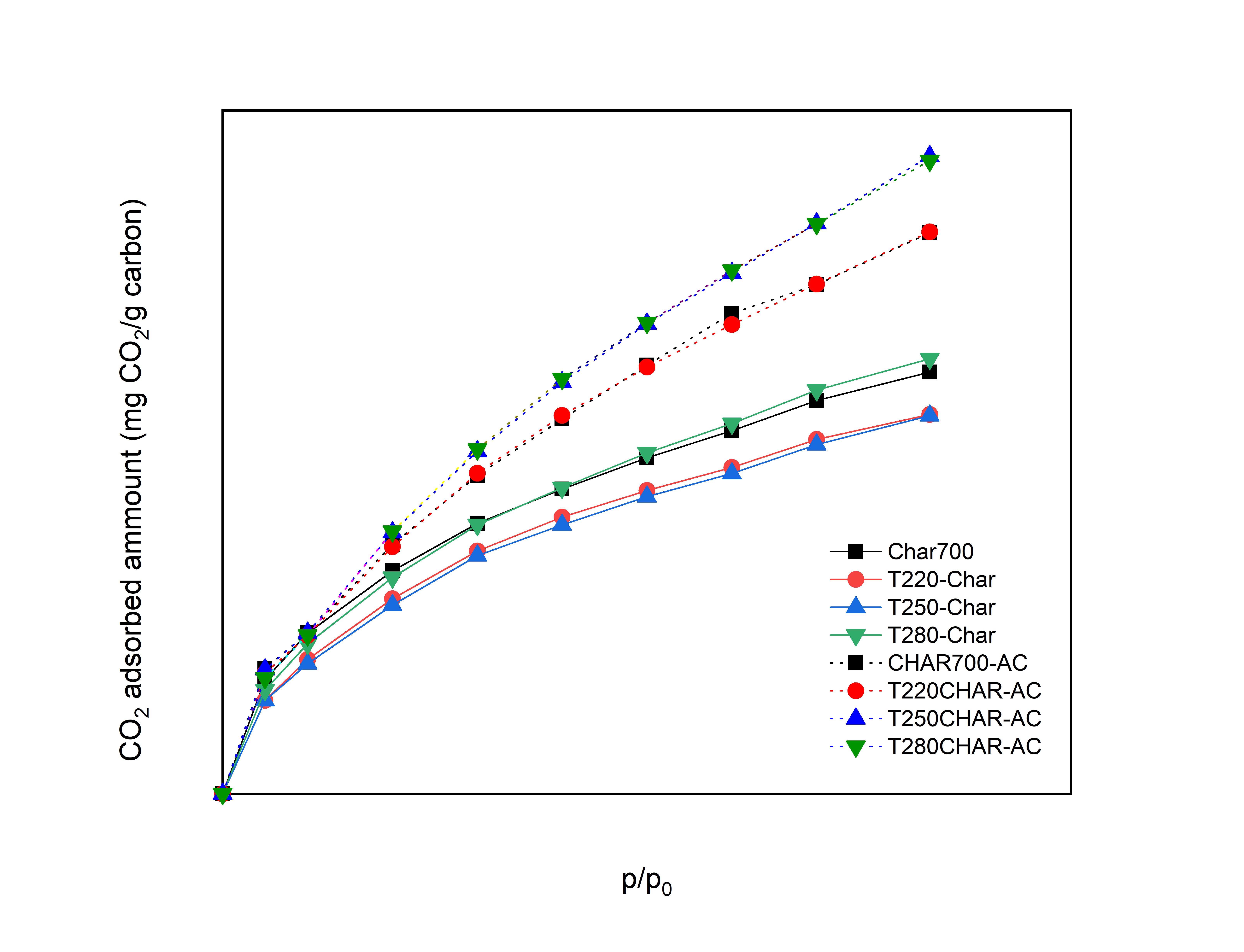
**

**Table S2.** Langmuir and Freundlich parameters obtained from CO_2_ adsorption isotherms at 298 K of biochar and activated carbon from raw palm kernel shell.

| **Sample** | **Freundlich** | | | **Langmuir** | | |
| --- | --- | --- | --- | --- | --- | --- |
|  | **R^2^** | **k_f_** | **n** | **R^2^** | **C_m_** | **K_g_** |
| Char700 | **0,9962** | 76,82 | 2,10 | **0,9697** | 69,44 | 6,86 |
| T220-CHAR-700 | **0,9978** | 68,85 | 2,00 | **0,9811** | 64,10 | 5,78 |
| T250-CHAR700 | **0,9987** | 67,60 | 2,00 | **0,9732** | 62,11 | 5,96 |
| T280-CHAR700 | **0,9993** | 78,07 | 1,96 | **0,9794** | 72,46 | 5,75 |
| CHAR700-AC | **0,9953** | 100,14 | 1,80 | **0,9435** | 89,29 | 5,33 |
| T220CHAR-AC | **0,9964** | 100,62 | 1,77 | **0,9516** | 90,91 | 5,00 |
| T250CHAR-AC | **0,9948** | 113,69 | 1,66 | **0,9459** | 103,09 | 4,41 |
| T280CHAR-AC | **0,997** | 115,55 | 1,60 | **0,9641** | 108,70 | 3,83 |

**ACKNOWLEDGMENT**

The authors thanks to the project "Transformation Strategy of the Colombian Energy Sector in Horizon 2030" funded by the call 788 for scientific ecosystems of Colciencias, contract number FP44842-210-2018 and the network "Alliance for Biomass and Sustainability Research (ABISURE), Universidad Nacional de Colombia", Hermes code 53024 for their support to conduct this study. The author Marlon Cordoba-Ramirez also appreciates the support provided by the Universidad de La Guajira to conduct this research, and the General Service of Support to Investigation -SAI- of University of Zaragoza for their collaboration with the FE-SEM analysis.
